# RNA Sequencing Reveals circRNA Expression Profiles in Chicken DF1 Cells Infected with H5N1 Influenza Virus

**DOI:** 10.3390/ani12020158

**Published:** 2022-01-10

**Authors:** Li Chen, Guoqin Li, Yong Tian, Tao Zeng, Wenwu Xu, Tiantian Gu, Lizhi Lu

**Affiliations:** Institute of Animal Husbandry and Veterinary Medicine, Zhejiang Academy of Agricultural Sciences, Hangzhou 310021, China; chenli0429@163.com (L.C.); ligq@zaas.ac.cn (G.L.); tyong@zaas.ac.cn (Y.T.); zengtao4009@126.com (T.Z.); xuwenwu248@outlook.com (W.X.); gtt19931029@126.com (T.G.)

**Keywords:** RNA-seq, H5N1, chicken, circRNA, DF1

## Abstract

**Simple Summary:**

H5N1 is a highly pathogenic avian influenza virus that seriously harms the poultry industry and public health worldwide. However, its pathogenesis is still not well understood. In this study, we analyzed the expression profile of circular RNAs (circRNAs) in H5N1-infected chicken embryo fibroblast (DF1) cells and found their expression to change more significantly as the infection was extended. Differentially expressed circRNAs were significantly enriched in terms relating to virus replication and immune response, suggesting that circRNAs play important roles in the pathogenesis of H5N1 infection. Our study provides new insights into the mechanisms underlying H5N1–host interaction.

**Abstract:**

H5N1, a highly pathogenic avian influenza virus that is prevalent in Asia, seriously harms the poultry industry and global public health. However, its pathogenesis is still not well understood. Circular RNAs (circRNAs), a newly identified type of RNA, reportedly play crucial roles in various pathogenic processes. In this study, RNA sequencing was performed to analyze the expression profile of circRNAs in H5N1-infected chicken embryo fibroblast (DF1) cells. A total of 14,586 circRNAs were identified. The expression profiles of infected cells changed more significantly, relative to uninfected cells, as the infection period was extended; namely, 261, 626, and 1103 circRNAs exhibited differential expression in cells infected for 6 h, 12 h, and 20 h, respectively. GO and KEGG enrichment analysis revealed significant enrichment of the parental genes of the differentially expressed circRNAs for viral replication and immune response-related pathways, such as positive regulation of transcription from the RNA polymerase II promoter, positive regulation of I-kappaB kinase/NF-kappaB signaling, innate immune response, and ubiquitin protein ligase activity. In conclusion, we identified the expression profile of circRNAs in H5N1-infected chicken DF1 cells. Bioinformatic analyses of the dysregulated circRNAs suggest that circRNAs might play important roles in the pathogenesis of H5N1 infection, offering new insights into the mechanisms underlying H5N1–host interaction.

## 1. Introduction

Influenza viruses belonging to the family Orthomyxoviridae are serious clinical and veterinary pathogens that cause epithelial cells to produce enveloped pleomorphic virions [1]. Influenza viruses can be classified into three types, A, B, and C, based on their major antigenic differences. Avian influenza virus (AIV) is of the A type, and is an important zoonotic pathogen [2] that can cause serious epidemics in poultry and result in substantial economic losses. Poultry infected with AIV exhibit reduced egg production, loss of appetite, soft or misshapen eggs, and even diarrhea and sudden death [3]. The first documented cases of human death caused by avian influenza virus infection were due to H5N1, a highly pathogenic avian influenza virus (HPAIV) first detected in Hong Kong in 1997 [4,5]. The H5N1 virus outbreak had devastating effects on the poultry industry, and the high pathogenicity of the virus provoked worldwide concern, but the pathogenesis underlying its infection is not yet fully clear.

Circular RNAs (circRNAs) comprise a novel category of RNAs with a “back-splicing” structure that confers remarkable tolerance to exonucleases [6,7]. CircRNAs are generated by the back-splicing of exonic, intronic, or intergenic regions. With the advance of high-throughput RNA sequencing (RNA-seq) technology, circRNAs were shown to be produced from thousands of loci in eukaryotes, from plants, animals to human beings [8,9], and were demonstrated to participate in multiple biological processes [10,11]. In recent years, they have been found to contribute substantially to host–virus interactions, specifically participating in the process of viral infection and the antiviral immune response. Transfection of circRNAs into mammalian cells can induce an innate immune response and confer protection against viral infection [12]. Lu et al. found that in cells infected with the Hantaan virus (HTNV), circ_0000479 regulated RIG-I expression by sponging miR-149-5p, thereby inhibiting viral replication [13]. Similarly, an artificial, designed circRNA was demonstrated to inhibit viral protein production in the HCV cell culture system by effectively sequestering cellular miR-122 [14], and abnormally expressed circRNAs in patients with chronic HBV infection may participate in immune regulation by regulating related miRNAs and their target genes [15]. However, while accumulated evidence has demonstrated substantial roles of circRNAs in viral pathogenesis, their particular roles in the pathogenesis of H5N1 remain largely unknown.

In this study, we used RNA-seq to investigate the expression profile of circRNAs and their potential roles in H5N1-infected chicken DF1 cells. Identifying the roles of dysregulated circRNAs could reveal their contributions to the interaction between H5N1 and the host. Our results provide novel insights into the mechanisms underlying H5N1 pathogenesis.

## 2. Materials and Methods

### 2.1. Cell Culture and Viral Infection

Chicken embryo fibroblast (DF1) cells obtained from the cell bank of the Chinese Academy of Agricultural Sciences were cultured in DMEM (Gibco) supplemented with 10% fetal bovine serum and 1% double antibody at 37 °C and 5% CO_2_. The highly pathogenic H5N1 strain A/wild duck/Huadong/S/2005 (SY) was propagated in ten-day-old non-specific pathogenic embryonic eggs. Viral titers were determined using the Reed and Muench method [16]. DF1 cells were infected at 0.01 multiplicity of infection (MOI) with H5N1 avian influenza virus for 6, 12, or 20 h, and uninfected DF1 cells were used as the control group. Three biological replicates were performed for each group. All live virus experiments were carried out in biosafety cabinets with HEPA filters in the Biosafety Level III Laboratory of Yangzhou University.

### 2.2. RNA Sequencing

Total RNA was isolated using TRIzol reagent (Life Technologies, Grand Island, NY, USA), and rRNA was subsequently removed from the total RNA using a Ribo-Zero Magnetic Kit (Epidemiology) (Epicentre). Next, RNA from each biological replicate was randomly broken into small fragments of about 200 bp, and cDNA libraries were constructed according to the instructions of the Illumina kit (Illumina, San Diego, CA, USA). Subsequently, paired-end sequencing with 150-bp read length was performed using the Illumina HiSeq 4000 platform. The sequencing data were submitted to the Genome Sequence Archive (GSA, https://ngdc.cncb.ac.cn/gsa/, accessed on 28 December 2021) with the accession number CRA005692.

### 2.3. Sequencing Data Analysis

To improve the reliability of circRNA identification, two programs, find_circ (v1.2) [17] and CIRCexplorer2 [18], were used to identify circRNAs, with their suggested settings. After removing low-quality reads, clean reads were mapped to the chicken reference genome (http://www.ensembl.org/Gallus_gallus/Info/Index, accessed on 28 December 2021) using the designated reads aligner recommended by the corresponding circRNA identification program. Subsequently, the sequencing data that could not be mapped to the reference genome directly were subjected to detection of back-splice junctions for circRNA annotation using the find_circ (v1.2) and CIRCexplorer2 programs with their default parameters. CircRNAs predicted by both programs were considered as candidate circRNAs for further analysis. The limma package (v3.32.10) [19] was used to identify the differentially expressed circRNAs (DE circRNAs). CIRCexplorer2 software was also used to annotate the parental genes of the circRNAs [18].

### 2.4. GO and KEGG Pathway Analysis

To analyze the functions of the differentially expressed circRNAs (DE circRNAs), we performed Gene Ontology (GO) and Kyoto Encyclopedia of Genes and Genomes (KEGG) pathway enrichment analyses on the parental genes of the DE circRNAs using the Database for Annotation Visualization and Integrated Discovery (DAVID, version 6.8; https://david.ncifcrf.gov/, accessed on 28 December 2021) [20] and the KEGG database (http://www.genome.jp/kegg/, accessed on 28 December 2021) [21].

## 3. Results

### 3.1. Characteristics of circRNAs

**Figure 1:**
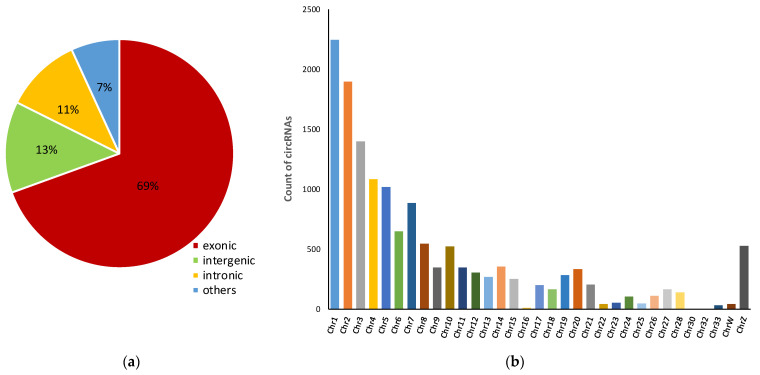
Characteristics of circRNAs. (**a**) Breakdown by circRNA type; (**b**) chromosome distribution of all identified circRNAs.

To identify the circRNA expression profile of DF1 cells infected with the highly pathogenic H5N1 avian influenza virus, we performed circRNA sequencing using rRNA-depleted total RNA from uninfected DF1 cells, as well as DF1 cells infected for 6 h, 12 h, and 20 h. A total of 14,586 circRNAs were identified. Exonic-derived circRNAs were the major type, accounting for about 69% of all detected circRNAs, while intergenic- and intronic-derived circRNAs only accounted for 13% and 11%, respectively (Figure 1a). We found circRNAs to be widely distributed across chromosomes, with the greatest number of circRNAs being located on chromosome 1 (Figure 1b). The distribution pattern of circRNAs across chromosomes is perfectly consistent with the chromosome length of chickens.

### 3.2. Identification of Differentially Expressed circRNAs

**Figure 2:**
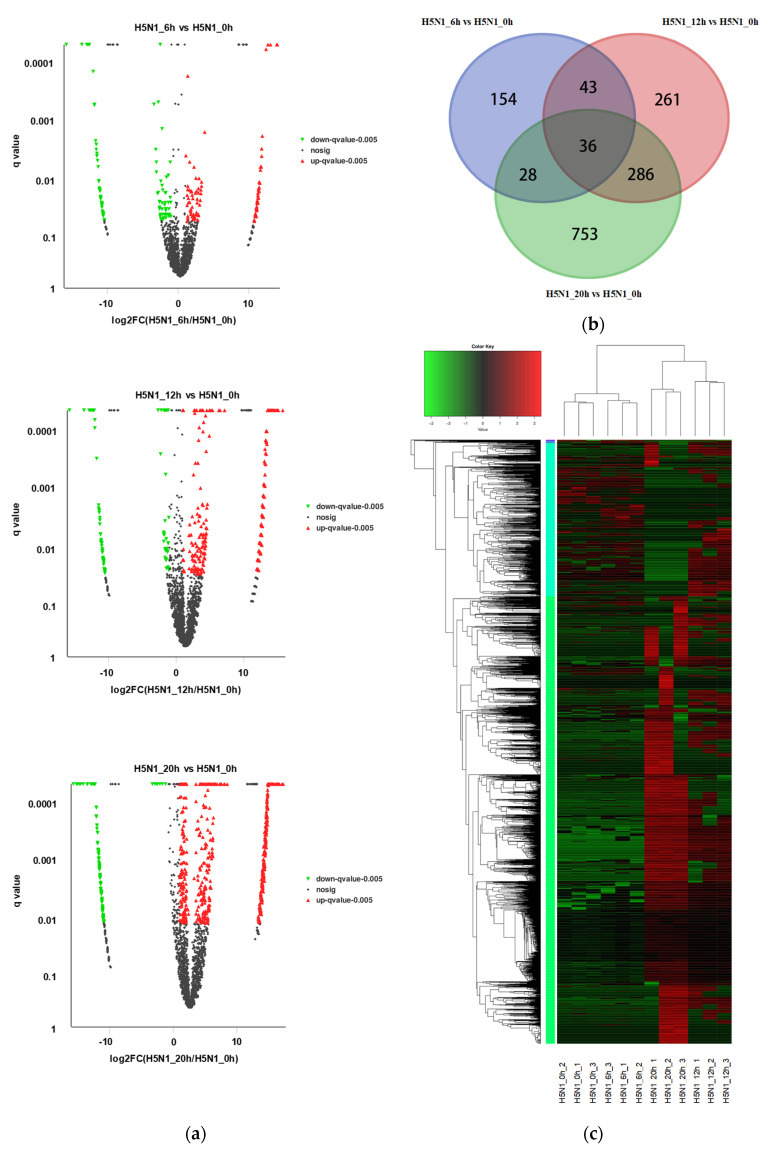
CircRNA expression changes in chicken DF1 cells infected with H5N1 avian influenza virus. (**a**) Volcano plot of circRNA expression. X–axis represents log2 (fold change) of circRNA expression; Y-axis represents q-value of circRNA expression changes. Red dots represent significantly upregulated circRNAs and green dots represent significantly downregulated circRNAs; (**b**) Venn diagram showing overlap of the differentially expressed circRNAs among timepoints; (**c**) hierarchical clustering heatmap of the DE circRNAs. Red represents high expression and green represents low expression. H5N1_0h, H5N1_6h, H5N1_12h, and H5N1_20h represent the uninfected group, 6-h infected group, 12-h infected group, and 20-h infected group, respectively.

CircRNAs with fold changes ≥ 2.0 relative to the uninfected group and *p*-values ≤ 0.05 were considered to be significantly differentially expressed. Under this definition, 261, 626, and 1103 circRNAs, respectively, exhibited differential expression after 6 h, 12 h, and 20 h of infection (Figure 2a). Of the differentially expressed circRNAs (DE circRNAs), 36 showed differential expression across all comparison pairs (Figure 2b). These circRNAs were annotated to 21 parental genes, of which seven had functions relating to immune response or virus infection, such as *CD2AP*, *QKI*, and *AKIRIN2* (Table 1). Hierarchical cluster analysis of all of the DE circRNAs revealed that circRNAs exhibited different expression patterns after different infection durations. That is, circRNA expression profiles could be used to classify samples into branches consistent with infection duration (Figure 2c). Overall, circRNA expression increased with infection time and was highest at 20 h of infection.

### 3.3. Functional Analysis of the Parental Genes of DE circRNAs

To identify the biological functions of the DE circRNAs in H5N1 infection, both GO and KEGG enrichment analyses were performed for the parental genes of the DE circRNAs. GO annotation analysis revealed significant enrichment of the viral replication-related categories, including “positive regulation of transcription from RNA polymerase II promoter” and “regulation of transcription from RNA polymerase II promoter” in the parental genes of the DE circRNAs for all H5N1 infected groups (Table S1). It is very interesting that immune response-related categories started to appear in the 12-h infected groups and increased in the 20-h infected groups. As shown in Table S1, the parental genes in the 12-h infected cells were enriched for immune response-related categories, including “positive regulation of I-kappaB kinase/NF-kappaB signaling” and “ubiquitin-protein transferase activity”, but the 20-h infected groups exhibited more immune response-related categories, such as “positive regulation of I-kappaB kinase/NF-kappaB signaling”, “negative regulation of NF-kappaB import into nucleus”, “innate immune response”, and “ubiquitin protein ligase activity”. KEGG pathway enrichment analysis, likewise, showed that a large number of parental genes of the 20-h DE circRNAs were principally enriched in immune response-related pathways, such as the “MAPK signaling pathway”, “Endocytosis”, “Ubiquitin mediated proteolysis”, and “Herpes simplex infection” (Table 2).

## 4. Discussion

Infection of chicken cells by the avian influenza virus results in dysregulation of the host transcription program. Large numbers of genes have been shown to be differentially expressed upon influenza virus infection [22,23,24]. Furthermore, infection activates many immune related-pathways, including the RIG-I pathway and the TLR, MAPK, TGF-beta, and NF-κB signaling pathways [22]. Beyond the alteration of messenger RNA transcription in influenza virus-infected cells, recent evidence has shown that circRNAs play crucial roles in the host response to influenza virus infection. In the current investigation, we identified 14,586 circRNAs. Consistent with current studies [25,26], exonic-derived circRNAs comprise the majority of identified circRNAs, compared with types of circRNAs arising from intronic and intergenic regions. Of the identified circRNAs, hundreds of differentially expressed circRNAs were identified in chicken DF1 cells infected with the H5N1 avian influenza virus, suggesting that circRNA expression is affected by the influenza virus and may participate in the pathogenesis of H5N1 infection. However, it is worth noting that these circRNAs need to be experimentally validated to exclude false positives.

circRNAs are posited to function in pathological conditions, such as virus infection, due to their potential to form competitive endogenous RNA (ceRNAs) that regulate innate immune responses [11]. There is also evidence that viral genomes could encode circRNAs that might function in viral infection [27,28]. Regarding particular known involvements of circRNAs, a study by Li and colleagues showed that activation of the innate immune sensor PKR induced nuclear export of NF90/NF110, which has promotive roles in circRNA biogenesis to inhibit viral infection [29]. In mice, infection with the H7N9 influenza virus induced differential expression of hundreds of circRNAs that play immune regulatory roles [30]. There is also evidence that host circRNAs were utilized to facilitate virus replication. For example, the human circRNA_0050463 facilitates IAV replication through sponging miR-33b-5p to regulate EEF1A1 [31]. The differentially expressed circRNAs in chicken DF1 cells infected with the H5N1 avian influenza virus were significantly involved in viral replication-related pathways, such as positive regulation of transcription from the RNA polymerase II promoter, and immune response-related pathways, such as positive regulation of I-kappaB kinase/NF-kappaB signaling, innate immune response, and ubiquitin protein ligase activity, suggesting that the H5N1 virus might escape or change the host’s immune response through affecting circRNA expression. These findings suggest that circRNAs might participate in the pathogenesis of H5N1 infection. Our study provides basic information regarding the transcription dynamics of circRNAs upon avian influenza virus infection; it would be helpful for a future study to conduct functional validation of the roles of circRNAs in AIV infection.

## 5. Conclusions

H5N1 is a highly pathogenic avian influenza virus that seriously harms the poultry industry and public health worldwide. In this study, we identified the expression profile of circRNAs in H5N1-infected chicken DF1 cells and found hundreds of circRNAs to be differentially expressed. The differentially expressed circRNAs were significantly enriched in terms relating to virus replication and immune response, suggesting that circRNAs might play important roles in the pathogenesis of H5N1 infection. Our study provides new insights into the mechanisms underlying the H5N1–host interaction.

## Figures and Tables

**Table 1:** Parental genes of the 36 common differentially expressed circRNAs. Genes relating to viral infection or immune response were annotated and highlighted in boldface.

CircRNA	Regulation	Gene Symbol	Terms/Pathways	Annotated Database
CIRI_circ_006230	down	-		
CIRI_circ_0013435	down (6:0); up (12:0; 20:0)	*ALKBH8*		
CIRI_circ_0011785	down	-		
**CIRI_circ_007242**	down	***RPL14***	**Influenza virus RNA transcription and replication.**	**Gene cards**
CIRI_circ_002201	down	-		
CIRI_circ_008390	down	-		
CIRI_circ_006022	down	-		
**CIRI_circ_008353**	down	***CUX1***	**Component of nf-munr repressor; binds to the matrix attachment regions (MARs) of the immunoglobulin heavy chain enhancer. Represses T-cell receptor beta enhancer function by binding to MARbeta.**	**UniProtKB/Swiss-Prot**
CIRI_circ_008531	up	*COL3A1*		
**CIRI_circ_0011293**	down	***CANX***	**Influenza virus RNA transcription and replication, and the innate immune system.**	**UniProtKB/Swiss-Prot**
CIRI_circ_003013	down	*COL3A1*		
CIRI_circ_008959	down	-		
CIRI_circ_002330	up	*AZIN1*		
CIRI_circ_0095	down (6:0); up (12:0; 20:0)	-		
CIRI_circ_005370	down	-		
CIRI_circ_007453	up	-		
CIRI_circ_00136	down (6:0; 12:0); up (20:0)	-		
CIRI_circ_004926	up	*TTC28*		
**CIRI_circ_005366**	up	***AKIRIN2***	**Required for the innate immune response.**	**UniProtKB/Swiss-Prot**
CIRI_circ_0013703	up	*MAFG*		
**CIRI_circ_003547**	up	***RPS6KA5***	**Activates TLR4 signaling and CNTF signaling.**	**UniProtKB/Swiss-Prot**
CIRI_circ_003861	down	*VNN2*		
CIRI_circ_00491	down (6:0); up (12:0; 20:0)	*DROSHA*		
CIRI_circ_0013304	down	*IGF1R*		
CIRI_circ_0014096	down	-		
CIRI_circ_004792	down	*TCP1*		
CIRI_circ_0010535	down	-		
CIRI_circ_003044	up	*ATRNL1*		
CIRI_circ_008077	up	-		
**CIRI_circ_0012305**	up	***CD2AP***	**May play a role in receptor clustering and cytoskeletal polarity in the junction between T-cell and antigen-presenting cell.**	**UniProtKB/Swiss-Prot**
CIRI_circ_008738	down	*LARGE1*		
CIRI_circ_002188	up	*DNAJB6*		
**CIRI_circ_007562**	up	***QKI***	**HIV Life Cycle and Oncogenic MAPK signaling**	**GeneCards**
CIRI_circ_002400	up	*TMEM214*		
CIRI_circ_009497	up	*MPP6*		
CIRI_circ_0014262	down	-		

**Table 2:** KEGG pathway enrichments of the parental genes of the circRNAs differentially expressed during H5N1 infection.

Group	Enriched Pathways	Enriched Genes	*p*-Value
6-h infected group	Dorsoventral axis formation	3	3.40 × 10^−2^
	Focal adhesion	7	3.50 × 10^−2^
	Wnt signaling pathway	5	7.40 × 10^−2^
	FoxO signaling pathway	5	7.60 × 10^−2^
12-h infected group	Focal adhesion	11	1.20 × 10^−2^
	Adherens junction	6	2.20 × 10^−2^
	SNARE interactions in vesicular transport	4	3.10 × 10^−2^
	Tight junction	6	3.40 × 10^−2^
	Cell cycle	7	4.60 × 10^−2^
	Ribosome biogenesis in eukaryotes	5	7.10 × 10^−2^
	ECM–receptor interaction	5	9.50 × 10^−2^
20-h infected group	MAPK signaling pathway	20	8.60 × 10^−4^
	Oocyte meiosis	12	8.90 × 10^−4^
	Endocytosis	17	1.60 × 10^−2^
	Focal adhesion	15	1.80 × 10^−2^
	Adherens junction	8	2.00 × 10^−2^
	Ubiquitin mediated proteolysis	11	2.80 × 10^−2^
	Regulation of actin cytoskeleton	14	2.80 × 10^−2^
	Progesterone-mediated oocyte maturation	8	3.30 × 10^−2^
	Hedgehog signaling pathway	4	5.20 × 10^−2^
	FoxO signaling pathway	10	5.60 × 10^−2^
	mRNA surveillance pathway	7	6.20 × 10^−2^
	Herpes simplex infection	11	6.20 × 10^−2^
	Adrenergic signaling in cardiomyocytes	9	8.70 × 10^−2^

## Data Availability

Not applicable.

## Supplementary Materials

The following are available online at https://www.mdpi.com/article/10.3390/ani12020158/s1, Table S1: GO analysis of the parental genes of circRNAs differentially expressed during H5N1 infection.
